# Polyethylene microplastic can adsorb phosphate but is unlikely to limit its availability in soil

**DOI:** 10.1016/j.heliyon.2023.e23179

**Published:** 2023-12-17

**Authors:** T.F. Khan, M.E. Hodson

**Affiliations:** a Environment and Geography Department, University of York, York, YO10 5NG, UK; b Department of Soil, Water and Environment, University of Dhaka, Dhaka 1000, Bangladesh

**Keywords:** Microplastic, Soil, Phosphate, Adsorption, Desorption

## Abstract

In plant growth experiments, the presence of microplastics (MPs) often reduces plant growth. We conducted laboratory experiments to investigate the potential of microplastics to adsorb the major soil nutrient phosphate; adsorption to MPs was then compared to adsorption to soil. Adsorption experiments used two contrasting soils, pristine high density polyethylene and artificially weathered material (the same material but exposed to 185 nm UV light for 420 h over 105 days), phosphate solutions (dissolved KH_2_PO_4_) ranging from 0.2 to 200 mg L^−1^ and a solid (g) to liquid (mL) ratio of 1: 150 at different values of pH (2–12) and different concentrations of background electrolyte (0.00–0.10 M NaNO_3_). The adsorption data were best fitted to linear and Freundlich isotherms. In initial experiments where pH was not fixed and with a background electrolyte of 0.10 M NaNO_3_, Kd values ranged from 3.37 to 27.65 L kg^−1^, log Kf from 1.21 to 1.96 and 1/n from 0.36 to 0.84. Exposure of the MP to 185 nm UV radiation led to the appearance of a C=O functional group in the MP; the partition coefficient Kd, calculated from the linear isotherm did not increase but the logKf value derived from fits to the Freundlich isotherm increased by a factor of 1.5. Kd values for soils were 3–7.5 times greater than those for MPs and log Kf values 1.1–1.7 greater. In the experiments in which initial pH and ionic strength were varied, adsorption was similar across all treatments with adsorption parameters for the higher organic content soil sometimes having the highest values and the pristine microplastic the lowest. In the desorption experiments most of the adsorbed phosphate desorbed. Overall our findings indicate that despite their ability to adsorb phosphate, MPs are unlikely to control the fate and behaviour of phosphate in soil.

## Highlights

• Weathered microplastics (MPs) adsorb more phosphate than pristine MP.
• Phosphate adsorption to MPs is best described by linear and Freundlich isotherms.
• Soils can adsorb more phosphate than MPs on a per mass basis.
• Phosphate sorption to MP is reversible.
• Sorption by MPs is unlikely to limit P availability or plant growth in soil.

## 1 Introduction

Microplastic (MP) pollution, its sources, global extent and impact, continues to receive much attention [1]. Although less widely reported than marine MP pollution, MPs may accumulate in terrestrial environments from a diverse range of sources, such as laundry dust, paint flakes, car tyres, sewage sludge, landfills and agricultural plastic sheeting [2,3]. Although it is now documented that MPs can have potentially negative effects on marine ecosystems [[4], [5], [6]], annual production of plastics continues to increase (e.g. from 359 million tons in 2018 to 368 million tons in 2019 [7]. Polyethylene (PE) is the dominant polymer produced in Europe, accounting for 29.8 % of total plastic production [7]. Commercial grade high-density polyethylene (HDPE) is lightweight, cheap and weather resistant; it is widely used in industries and packaging sectors. HDPE is suitable for a wide range of applications from heavy-duty damp proof membranes to light, flexible carrier bags and films due to the unique characteristics of HDPE [8,9].

Once in the environment, UV radiation, elevated temperature, abrasive/mechanic force and microbial activities can lead to the fragmentation of HDPE and the formation of MP particles [10,11], which can adsorb metals [[12], [13], [14]] and organic compounds [[15], [16], [17]]. Studies on the adsorption of anions to MPs are rare; the adsorption of oxyanions of Cr [18,19] and As [20,21] has been observed. Similarly, reductions in As uptake by plants in the presence of MPs are consistent with adsorption of oxyanions of As by the MPs reducing their bioavailability [22,23]. We are not aware of comparable studies on the adsorption of anionic plant nutrients on MPs. MPs can reduce plant growth [[24], [25], [26], [27]]. Although the underlying reasons for this are not clear a possible reason is nutrient immobilisation. Studies to understand the interactions between MPs and plant nutrients are therefore warranted.

Phosphorus is a key plant nutrient that occurs in soil; reductions in available phosphate in soil can occur if phosphate is irreversibly adsorbed to soil components and can limit plant growth [28]. Phosphate adsorption onto soil components is dominated by Fe oxides, e.g. [29,30] though clay minerals also contribute binding sites, e.g. [31] and increases in P adsorption with organic matter content have been reported, e.g. [[32], [33], [34], [35], [36], [37]]. Adsorption typically decreases with increasing pH, [[38], [39], [40]] though this is not always the case and is a function of soil pH, organic matter content and potentially other properties as well, e.g. [34]. Reported responses to increases in ionic strength are mixed with adsorption being observed to both increase, show no change or decrease depending on pH and adsorption mechanism [38,39,41]. To the best of our knowledge, to date no one has explored the effects of pH and concentration of the background electrolyte on phosphate adsorption to MP.

The aims of the present experiment were therefore to determine: (1) the potential for MPs generated from HDPE powder to adsorb phosphate compared to soil; (2) whether this sorption was reversible; (3) whether UV exposed MP adsorbs more phosphate than pristine MP; and (4) whether pH and concentration of the background electrolyte have impacts on the adsorption of phosphate to the MP.

## 2 Material and methods

### 2.1 Materials

Two types of soil were selected for use in the experiments to provide soils with contrasting organic matter and Fe oxide contents. Soil 1 (S1) was collected from an arable field (U.K. grid references 53° 52′ 25.2″ N, 1° 19′ 47.0″ W) located at the University of Leeds experimental farm; a higher organic matter soil (S2) was collected from a flower bed (53° 56′ 41.0″ N, 1° 3′ 04.9″ W) located on the University of York campus. The soils were air-dried and sieved to <2 mm prior to characterisation. Texture [42], pH [43], organic matter content [43,44], dithionite-citrate extractable Fe as a proxy for amorphous and organically-complexed Fe [45] and point of zero charge (PZC) [46] of both soils were determined following standard methods (details in Supporting Information, SI). S1 had a silty loam texture, a pH of 7.63 ± 0.13, an organic matter content of 3.58 ± 0.15 %, a dithionite-citrate extractable Fe content of 15.0 ± 1.17 mg kg^−1^ and a PZC of 5.27 ± 0.08, whereas S2 had a loamy texture, a pH of 7.11 ± 0.09, an organic matter content of 4.62 ± 0.13 %, a dithionite-citrate extractable Fe content of 3.22 ± 0.07 mg kg^−1^ and a PZC of 4.51 ± 0.06 (n = 3, ±standard deviation for all measurements).

Commercial grade high-density polyethylene (HDPE) MP powder was purchased from Qingdao Sunsoar Tech. Co., Ltd. Located in China. Size and shape properties of the MP particles were determined using a Zeiss PlanNeoFluar Microscope at 33.5 magnification and ImageJ (v. 1.53) software [47] (Table S1). The MP was confirmed to be HDPE using Fourier Transform Infrared Spectroscopy (Bruker– Alpha FTIR, Germany) equipped with an ATR platinum diamond attenuated total reflectance accessory and a potassium bromide beam splitter (Fig. S2). Spectra were scanned in the range of 400–4000 cm^−1^. Each spectrum comprised of 144 scans with a 4 cm^−1^ resolution.

Weathering of the MPs was simulated by the use of a 185 nm UV lamp (Analytikjena PN 90-0019-01, USA) [48]. Approximately 10 g pristine MP (PMP) were placed in a 100 mL Erlenmeyer flasks in a 1 mm deep layer covering the bottom of the flask. There was a distance of five cm between the sample and the radiation source. The sample flask was wrapped in aluminium foil. The UV lamp was turned on automatically for 4 h every day over a period of 105 days. FTIR spectra were obtained every two weeks in the range of 400–4000 cm^−1^; each spectrum comprised 144 scans with a 4 cm^−1^ resolution. The PMP clumped together after exposure to the UV radiation whereas no clumping was observed beforehand (Fig. S3). To declump the weathered MP (WMP), 10 g WMP were submerged in a beaker of deionized water and agitated using an ultrasonic probe (MSE Soniprep 150 Plus). The WMPs were then left for 24 h at room temperature (22 ± 2 °C) for drying. The size and shape of the WMPs were determined using ImageJ software (Table S1).

### 2.2 Adsorption experiments

Adsorption experiments were carried out to determine the potential for MPs to adsorb phosphate and to compare the sorption capacity of PMP and WMP to that of soil. Initial kinetic experiments to determine appropriate experimental run times were carried out using 0.2 g of soil/MP or a mixture of 0.1 g soil and 0.1 g MP in 30 mL of 5 mg L^−1^ phosphate solution obtained by dissolving potassium dihydrogen phosphate (KH_2_PO_4_) in a background electrolyte of 0.10 M NaNO_3_. Samples were shaken at 180 rpm at 20 °C on a flatbed shaker [49]. Control vials, containing phosphate solution but without solids were treated the same way. After 1, 3, 6, 14, 24 and 48 h triplicate sacrificial replicates were filtered through Whatman No. 42 filter paper and the supernatants were analyzed for phosphorus content using an Inductively Coupled Plasma – Optical Emission Spectrometer (ICP-OES, Thermo Scientific ICAP 7000). Data indicated that adsorption had reached a steady state (no significant difference in concentration between the last three sampling points) within 14/24 h (Fig. S4). Therefore subsequent adsorption experiments were run for 24 h.

Adsorption experiments to construct isotherms were conducted in the same way as above for a duration of 24 h. Solutions of 0.2–200.0 mg L^−1^ phosphate were used; there were 10 nominal concentrations in total (0.2, 0.5, 1.0, 2.0, 5.0, 10.0, 20.0, 50.0, 100.0 and 200.0 mg L^−1^). Data were fitted to linear, Langmuir and Freundlich isotherms [50,51].

Adsorption experiments to investigate the effects of pH and concentration of the background electrolyte were carried out as above with solutions of 0.2–200.0 mg L^−1^ phosphate, but pH (2–12) and background electrolyte concentration (0.00–0.10 M NaNO_3_) were varied. Deionized water was used instead of NaNO_3_ in 0.00 M treatments. At each concentration of the background electrolyte, pH of the solution was adjusted by adding 0.05–0.50 M HNO_3_/NaOH until the pH was close to the desired value. pH was then allowed to drift over the course of the adsorption experiment [52].

### 2.3 Desorption experiments

Desorption experiments following the method of Han et al. [53] were undertaken to determine whether MPs could release previously sorbed phosphate and to compare desorption with that from soils. Solids from the initial adsorption experiments were collected on Whatman No. 42 filter paper and washed with 30 mL saturated NaCl to remove free P. Solids were then added to 30 mL of 0.10 M NaNO_3_. The mixtures were shaken at 180 rpm for 24 h, filtered using Whatman No. 42 filter paper and finally the supernatants were analyzed using ICP-OES to determine P concentration in the solution.

### 2.4 Quality control, data processing and statistical analysis

Quality control data associated with each set of experiments are provided in Table S2. Accuracy for P analyses ranged from 97 to 98 %, precision 0.39–2.38 % and detection limits from 0.05 to 0.77 mg L^−1^. To calculate adsorption onto solids, the average concentration in the triplicate solid-free controls was used as the initial concentration in solution. However, for convenience in the text where initial concentrations are cited, target rather than actual concentrations are used. Measured initial concentrations are presented in Table S3. For the kinetic experiments two-way analysis of variance (ANOVA) was carried out in SigmaPlot (version 14) to determine significant differences in phosphorus concentration between solid type and time with Tukey post hoc pair-wise analysis used to determine when there were no significant changes in concentration between time intervals. For the adsorption experiments the fit of the data to linear, Langmuir and Freundlich isotherms was determined by linear regression carried out using the Data Analysis ToolPak in Microsoft Excel 2016. The slopes and intercepts from the best-fit straight lines were used to determine linear partition coefficients (Kd), maximum adsorption capacities (Csm), binding constants (b), Freundlich constants (Kf) and heterogeneity factors (1/n). Values were deemed to be significantly different if the 95 % confidence intervals did not overlap [54]. For values of Kd the regression line was constrained to pass through the origin. Tests for normality and either Pearson or Spearman rank correlations, as appropriate, were carried out to assess variation of adsorption parameters with pH using SigmaPlot 15.

## 3 Results

### 3.1 UV weathering of the microplastic

The FTIR spectra of the pristine microplastic (PMP) showed distinct peaks at 2917 cm^−1^, 2847 cm^−1^, 1470 cm^−1^, 1440 cm^−1^, 750 cm^−1^ and 713 cm^−1^ (Table 1; Fig. S2), characteristic of PE. For the MP exposed to the 185 nm UV radiation (WMP), no changes in the spectra were observed up to 60 days of exposure. From day 75, a new peak was detectable at 1743 cm^−1^ (Table 1) which became increasingly visible up to day 105 (Fig. S5).

**Table 1:** Wave number and associated functional groups for peaks in the FTIR spectra of microplastic before and after exposure to UV radiation. * indicates the additional peak observed in the WMP spectra.

Wave number (cm^−1^)	Functional group	Appearance	References
2917	*C*–H stretching	Strong	[55,56]
2847	*C*–H stretching	Weak	[55,56]
1743*	C=O stretching	Strong	[57,58]
1470	CH_2_ bending	Medium	[[55], [56], [57]]
1440	CH_2_ bending	Medium	[55]
750	CH_2_ rocking	Strong	[[55], [56], [57]]
713	CH_2_ rocking	Strong	[55,56]

### 3.2 Adsorption in 0.1 M NaNO_3_ with unconstrained pH

Results for the 200 mg L^−1^ treatment indicated far lower adsorption than for the 2–100 mg L^−1^ results and therefore appear to be anomalous. The data are given in Table S3 but are not considered in the following analysis; it was not practical to repeat the 200 mg L^−1^ experiments. The adsorption data best fitted linear and Freundlich isotherm models, fits to the Langmuir isotherm were less good (Table 2). The PMP and WMP had similar Kds. Kd values were greater for the soils than the MPs with Kd for S2 being greater than that for S1. The Kds of the MP plus soil mixtures all had similar values to those of the MPs. PMP had a lower logKf than the other solids, values of 1/n showed no consistent trends.

**Table 2:** Linear, Langmuir and Freundlich isotherm fits and parameters for the different solids investigated with a background electrolyte of 0.1 M NaNO_3_ and uncontrolled pH. PMP = pristine PE, WMP = artificially weathered PE, S1 and S2 are soils, S1+PMP, S1+WMP, S2+PMP and S2+WMP are 50:50 wt % mixtures. Langmuir equation is expressed as $\frac{C_{s}}{C_{\text{aq}}}=\frac{bC_{\text{sm}}}{1+C_{\text{aq}}b}$, where C_s_ = amount of P adsorbed on solid (mg/kg), C_aq_ = concentration of P in equilibrium solution (mg L^−1^), C_sm_ = maximum adsorption capacity (mg kg^−1^), b = binding constant (L kg^−1^). Freundlich equation is expressed as Cs= Kf $C_{\text{aq}}^{\;1/n}$ where C_s_ = amount of P adsorbed on solid (mg kg^−1^), C_aq_ = concentration of P in equilibrium solution (mg L^−1^), log Kf = Freundlich constant, 1/n = heterogeneity factor.

Linear							
Solid type	Kd	95 % confidence interval	R^2^	p			
PMP	4.61	0.46	0.90	<0.01			
WMP	4.94	1.30	0.64	<0.01			
S1	15.09	3.14	0.75	<0.01			
S2	27.65	3.48	0.87	<0.01			
S1+PMP	3.47	0.43	0.87	<0.01			
S1+WMP	5.49	1.38	0.66	<0.01			
S2+PMP	3.37	0.36	0.89	<0.01			
S2+WMP	8.19	1.17	0.84	<0.01			
Langmuir							
Solid type	C_sm_	95 % confidence interval	b	Lower 95 % confidence interval	Upper 95 % confidence interval	R^2^	p
PMP	20.30	30.19	3.78	3.29	16.01	0.12	<0.05
WMP	124.40	90.29	1.02	−0.64	1.73	0.46	<0.01
S1	1046.26	125.90	0.09	−0.84	0.73	0.82	<0.01
S2	33.26	25.79	2.76	−3.36	3.59	0.18	<0.01
S1+PMP	73.78	209.90	7.06	6.82	7.64	0.46	<0.01
S1+WMP	63.24	82.08	7.01	6.76	9.76	0.15	<0.05
S2+PMP	109.08	331.54	1.53	1.16	1.83	0.92	<0.01
S2+WMP	332.73	76.89	0.34	−1.53	1.44	0.68	<0.01
Freundlich							
Solid type	log Kf	95 % confidence interval	1/n	95 % confidence interval	R^2^	p	
PMP	1.21	0.23	0.65	0.18	0.65	<0.01	
WMP	1.79	0.13	0.56	0.10	0.82	<0.01	
S1	1.96	0.15	0.66	0.14	0.78	<0.01	
S2	1.95	0.26	0.84	0.22	0.67	<0.01	
S1+PMP	1.70	0.12	0.36	0.09	0.71	<0.01	
S1+WMP	1.80	0.14	0.52	0.11	0.76	<0.01	
S2+PMP	1.64	0.09	0.42	0.07	0.85	<0.01	
S2+WMP	1.83	0.08	0.61	0.06	0.93	<0.01	

### 3.3 Effects of pH and concentration of the background electrolyte on adsorption

**Fig. 1:**
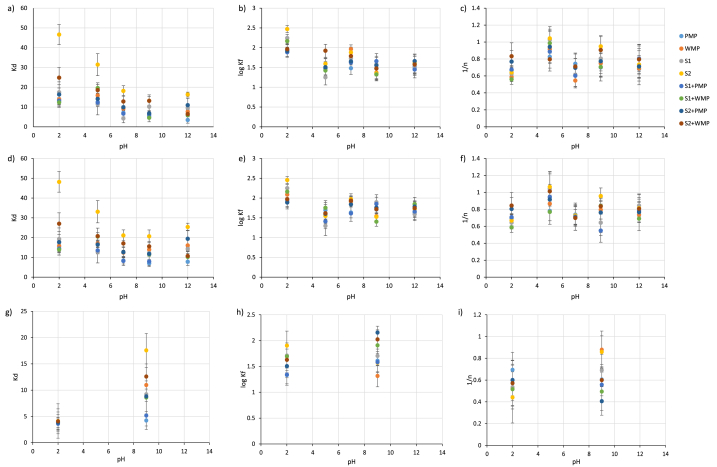
Adsorption isotherm fits for a – c) 0.00 M, d – f) 0.01 M and g – i) 0.10 M ionic strength solutions to a, d, g) linear isotherm Kd, b, e, h) Freundlich isotherm log Kf and c, f, i) Freundlich isotherm 1/n. PMP = pristine PE, WMP = artificially weathered PE, S1 and S2 are soils, S1+PMP, S1+WMP, S2+PMP and S2+WMP are 50:50 wt % mixtures. Error bars indicate 95 % confidence intervals.

As with the initial adsorption experiment, adsorption was best described by linear and Freundlich isotherms; some fits to the Langmuir isotherm returned negative values for maximum adsorption capacity (Table S3). Data for the 0.10 M ionic strength pH 5, 7 and 12 experiments yielded values of Kd 2 to 3 orders of magnitude greater than those obtained in the initial experiment and are therefore thought to be anomalous. The data are given in Table S3 but not considered in the following analysis; it was not practical to repeat these experiments. Typically Kd values were greatest for S2 and least for PMP; WMP and S1 had similar values (Fig. 1). At pH 2 Kd values were lower in the 0.1 M solution than in the 0.00 M and 0.01 M solutions. Log Kf values were similar across all treatments with S2 sometimes having the highest values and PMP the lowest. At pH 2 log Kf values were lower in the 0.10 M solution than in the 0.00 M and 0.01 M solutions whereas at pH 9 log Kf was highest in the 0.10 M solutions for the S1+WMP, S2+PMP and S2+WMP treatments. 1/n values were similar across all treatments. In the 0.00 M and 0.01 M ion strength solutions Kd either decreased with pH (PMP 0.00 M, 0.01 M, S2 0.00 M, S1+PMP 0.00 M, S2+WMP 0.00 M, 0.01 M, r = −0.89 to −0.99, p ≤ 0.05) or showed no consistent trend with little difference between values at the different pHs; log Kf and 1/n values showed no consistent trend with pH.

### 3.4 Desorption experiments

**Fig. 2:**
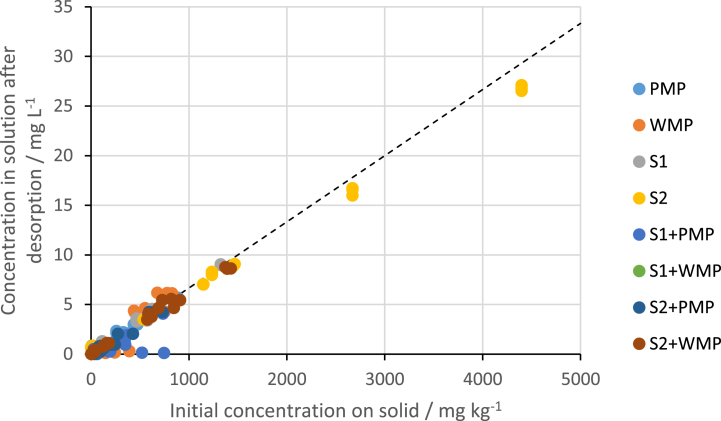
Concentrations of phosphate desorbed into solution when the solids used in the initial adsorption experiment were washed then transferred to phosphate free 0.10 M NaNO_3_ solution. The solid diagonal lines represents where data would plot if all the initially adsorbed phosphate was desorbed into solution. PMP = pristine PE, WMP = artificially weathered PE, S1 and S2 are soils, S1+PMP, S1+WMP, S2+PMP and S2+WMP are 50:50 wt % mixtures.

Desorption of phosphate from the solids used in the initial adsorption experiment was proportional to the amount that was initially adsorbed (Fig. 2) though at lower concentrations there is more deviation from a straight line relationship. The majority of phosphate that was initially adsorbed was desorbed.

## 4 Discussion

In our experiments microplastics adsorbed phosphate at all pHs (Fig. 1, Table 2). Adsorption of phosphate to the MPs (and soil) was better described by Freundlich and linear isotherms rather than Langmuir isotherms (Tables 2, S3). Whilst some literature studies also find this to be the case for the adsorption of inorganic cations and oxyanions (Cr ions) to MPs e.g. [12,18,59], in others adsorption of ions to MPs is better described by the Langmuir isotherm e.g. [60,61] or there is no difference in the fits, e.g. [13]. Similarly, for studies of P sorption to soil, good fits are seen to both Langmuir and Freunlich fits [32,33,37,49,62,63]; fits to linear isotherms are rarely considered. Which isotherm best describes the adsorption data seems to be highly dependent on the specifics of each system with little systematic variation. This is perhaps to be expected as isotherm fits are descriptions of concentration data rather than specific to particular reaction mechanisms [50]. The determined adsorption parameters had values similar to others reported in the literature for phosphate adsorption to soils and Fe oxides, e.g. [32,33,37,49,[63], [64], [65]].

Phosphate is present in solution as a range of anions, e.g. [30] whereas polyethylene is a non-polar compound comprising almost entirely [–CH_2_–CH_2_]_n_ units [66]. Similar to the adsorption of other ions, both cations and anions, by pristine microplastics, e.g. [[12], [13], [14],^18^,^59^,^60^,^67^], this suggests that in this case adsorption is dominated by weak van der waal forces rather than by either electrostatic attraction to charged surfaces or by ligand exchange. Polar C=O functional groups were detected in the WMP (Table 1). These can increase adsorption of charged ions onto otherwise hydrophobic surfaces, and, in common with other studies, e.g. [13,18,68] we saw evidence of increased adsorption onto the WMP relative to the PMP though the values overlapped when 95 % confidence intervals were taken into account. Adsorption onto the soils was greater than onto the MPs and was greater onto S2 than S1. Given the higher Fe oxide content of S1 and the dominant role of Fe oxides in P sorption in soils, e.g. [29,30] we had anticipated that S1 would exhibit greater adsorption for P than S2. As this is not the case, and as S2 has a higher organic matter content than S1 it perhaps suggests that in these soils metal bridging, e.g. [69,70] plays an important role in P sorption. In any event increased P sorption with increased organic matter content has been reported previously, e.g. [[32], [33], [34], [35], [36]], consistent with S2 being more P sorptive than S1. Unfortunately dissolved organic carbon measurements were not made in our experiments, nor was it feasible to carry out spectroscopic measurements which may have helped determine the predominant bonding mechanism. The mixtures of MPs and soils tended to show adsorption that was more similar to the MPs than the soils. The mixtures were established on a per mass basis but adsorption is a surface phenomenon. The surface area of the MPs and soils was not measured but given the size of the MPs (Table S1) and the texture of the soils it is likely that the specific surface area, i.e. surface area per unit mass, of the MPs was significantly greater than that of the soils. In that case the adsorption properties of the MPs would have had a greater influence on the mixtures than the soils.

In our experiment, adsorption showed little variation with pH but where it did vary it decreased with increasing pH. Similar trends have been observed by both Holmes et al. [18] and Zhang et al. [19] for Cr adsorption (which like phosphate is present as an anion in solution) on PE MPs whilst Wang et al. [61] and Zou et al. [59] observed an increase in the adsorption of cations on PE MPs with pH. This suggests an electrostatic component to the adsorption of ions to MPs, despite their non-polar composition. As pH increases surfaces can change from having a positive charge to a negative charge [50] leading to increased electrostatic attraction for cations and increased repulsion for anions. Additionally there will be less competition for positively charged sites between the ions of interest and H^+^ and more competition for negatively charged sites between the ions of interest and OH^−^ [61]. However, P sorption to soil has also been found to be independent of pH [34] and is a function of various soil parameters, such that it is hard to predict how pH is likely to impact sorption.

Various studies have observed decreases in adsorption of both oxyanions [18,60] and cations [59,61] on PE MPs with increases in ionic strength of solution. This has been variously attributed to enhanced aggregation reducing the surface area available for adsorption, competition between the electrolyte and ions of interest for sorption sites, reductions in ion activity and compression of the electrical double layer [59,61,71] and is suggestive of adsorption being controlled by electrostatic interactions. We saw little difference in adsorption between our 0.00 M and 0.01 M solutions but at pH 2 we did observed a reduction in adsorption with increased ionic strength. However, at pH 9 we observed an increase in adsorption. Studies on other soil components such as Fe oxides suggest that at lower pH values adsorption is unaffected or shows a decrease with increasing ionic strength whereas at higher pH values adsorption increases with increasing ionic strength [38,39,41,50] and attribute this to adsorption being dominated by ligand exchange at lower pH and electrostatic attraction at higher pH. Thus our data suggest a shift in the dominant sorption mechanism with pH but as mentioned above it was not possible to perform appropriate spectroscopic studies here that could have confirmed this.

The high level of desorption from both the MPs and soils is further evidence that adsorption was likely dominated by weak van der waal forces rather than electrostatic attraction or ligand exchange, even in the soils. Other studies have also found that metals adsorbed to MPs readily desorb, e.g. [61]. Given the relatively low concentrations of MPs found in soils, it also suggests that the presence of MPs in soils is unlikely to limit phosphate availability, even when some adsorption occurs.

## 5 Conclusions

Our study demonstrated that PE MPs can adsorb phosphate and that UV weathering increases the amount of adsorption. Adsorption was less than that observed in soils and was largely reversible. Given the relatively low concentration of MPs in soils it therefore seems unlikely that reduced plant growth in the presence of PE MPs is due to reduced phosphate availability. However, the sorptive capacity of MPs does vary between polymers and increases with decreased particle size and increased weathering, therefore it is not possible to rule out the possibility of a negative effect of MPs on soil nutrients entirely, particularly as MPs weather and decrease in size.

## Data availability statement

All data associated with study are present in the Excel files attached as Supplementary information.

## CRediT authorship contribution statement

**T.F. Khan:** Writing - original draft, Investigation, Funding acquisition, Formal analysis, Conceptualization. **M.E. Hodson:** Writing - review & editing, Supervision, Funding acquisition, Formal analysis, Conceptualization.

## Declaration of competing interest

The authors declare that they have no known competing financial interests or personal relationships that could have appeared to influence the work reported in this paper.
