# Supplementary material for: Polyethylene microplastic can adsorb phosphate but is unlikely to limit its availability in soil
Source: Heliyon. 2023 Dec 17;10(1):e23179. doi: 10.1016/j.heliyon.2023.e23179 (PMC10772576; doi:10.1016/j.heliyon.2023.e23179)
Supplement: Multimedia component 1 [file mmc1.docx]

**Supporting Information**

***Soil characterisation***

The soils were air-dried, visible roots and plant debris were discarded, and the soils were ground gently to break up larger soil aggregates. After that the soils were sieved at 2 mm, thoroughly homogenized and finally characterised.

Soil texture was determined manually (USDA, 1951). A ball of soil was placed between the thumb and forefinger, gently pushing the soil with the thumb and working it upward to make a ribbon. The ribbon was allowed to emerge and extend over the forefinger, breaking from its own weight. Finally the texture of the soil was determined from the length and strength of the ribbon.

The pH of the soil was determined by mixing air-dried soil and deionised water at a ratio of 1:2.5 followed by shaking for 15 minutes. The suspension was allowed to settle and pH was measured using an Accumet AB150 pH meter calibrated with pH 4.0, 7.0 and 10.0 buffers (Rowell, 1994).

Soil organic matter was determined by heating c. 20 g of air-dried soil overnight at 105 °C, reweighing and then heating at 350 °C overnight. Mass loss on ignition (LOI) was determined and used as a proxy for organic matter content (Hoogsteen et al., 2018; Rowell, 1994).

Dithionite-citrate extractable Fe extracted 0.5 g air-dry soil measured accurately to 3 D.P. with 25 mL of 200 g L^-1^ sodium citrate and c. 0.4 g sodium dithionite overnight. Method blanks were also run. The mixtures were centrifuged at 510 g for 20 minutes, filtered with Whatman 40 filter papers, diluted by a factor of c. 10 and analysed by ICP-OES for Fe and Al (Courchesne and Turmel, 2008). Solution detection limits were 0.098 and 0.043 mg L^-1^ which corresponded to c. 4.91 and 21.5 mg kg^-1^. Method blanks were below detection for Al but above detection for Fe (c. x100 lower in value than sample values) so Fe values were blank corrected. Precision was 2.5 % and 2.3 % for Fe and Al, accuracy was 98 % and 116%.

The point of zero charge was determined according to the mass titration method outlined by Noh and Schwarz (1989). In brief, the experiment was carried out using an increasing mass of the soils which were added to a known volume of KCl and allowed to equilibrate for about 24 hours to achieve optimal mixing and adsorption of ions onto the soils. Increasing mass of the soils were added to the centrifuge tubes containing 40 ml of 0.1 M KCl solution. The centrifuge tubes were shaken using the horizontal shaker (IKA, KS 260 basic, Europe) for 24 hours, centrifuged at 2000 rpm for 10 minutes and filtered through Whatman filter paper (#42). After that the equilibrium pH of each solution was measured by a pH meter (Benchtop, Thermo Orion). Equilibrium pH versus the amount of the solid added was plotted (Fig. S1), and finally the PZC was determined at the inflection point of the plateau.

Fig. S1. Determination of point of zero charge for a) PMP, b) WMP, c) S1, d) S2, e) S1+PMP, f) S1+WMP, g) S2+PMP and h) S2+WMP. Equilibrium pH versus the mass of the solid was plotted. PMP = pristine PE, WMP = artificially weathered PE, S1 and S2 are soils, S1+PMP, S1+WMP, S2+PMP and S2+WMP are 50:50 wt % mixtures.

***Microplastic characterisation***

The MP was confirmed to be HDPE using a Bruker Fourier Transform Infrared Spectrometer equipped with an ATR platinum diamond attenuated total reflectance accessory and a potassium bromide beam splitter. Spectra were scanned in the range of 400 to 4000 cm^-1^. Each spectrum comprised of 144 scans with a 4 cm^-1^ resolution. The ATR crystal of the instrument was cleaned with 70 % 2-propanol and a blank sample was analysed first to establish a background spectrum for background correction of subsequent spectra. Background spectrum showed a broad intense peak at 1575 and 1660 cm^-1^ representative of the H-O-H bond in water. Water was also represented by a peak at 3791 cm^-1^ corresponding to the O-H bond. No other peaks were observed in the background spectrum. When background measurement was completed, approximately 2 g of HDPE powder was placed onto the ATR crystal and spectra were obtained.


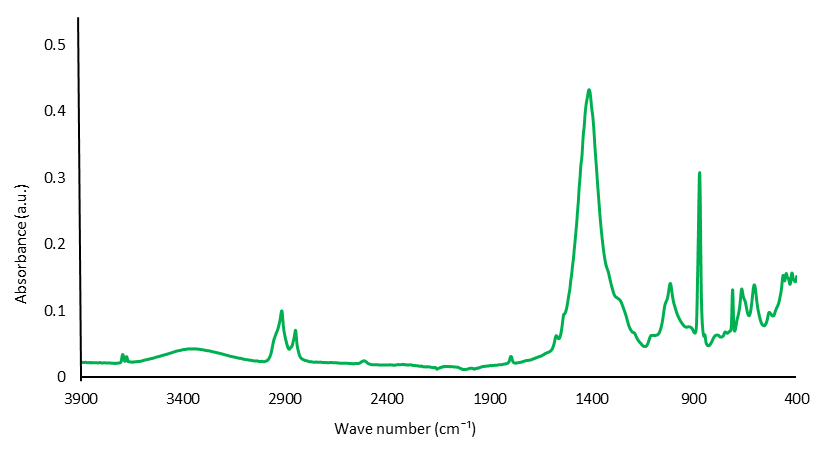


Fig. S2. FTIR spectra of pristine microplastic (PMP)

Fig. S3. MP particles a) before and b) after exposure to the UV radiation with 185 nm wavelength under microscope. Field of view in b) is 7mm. After exposure particles showed a tendency to clump together. Particles were scattered on a slide, flattened by over-laying a piece of glass and images captured with a Zeiss PlanNeoFluar Microscope.

Table S1. Properties of MPs (n = 300, ± standard deviation).

| Parameter | Untreated MP | MP exposed to 185 nm  UV light |
| --- | --- | --- |
| Average surface area (mm^2^) | 0.06 ± 0.01 | 0.2 ± 0.001 |
| Perimeter (mm) | 0.25 ± 0.50 | 0.28 ± 1.13 |
| Circularity | 0.96 ± 0.52 | 0.97 ± 0.71 |
| Roundness | 0.89 ± 0.33 | 0.91 ± 0.23 |
| Diameter (µm) | 238.11 ± 82.52 | 249.86 ± 38.61 |
| Major axis (mm) | 0.24 ± 0.09 | 0.25 ± 0.09 |
| Minor axis (mm) | 0.14 ± 0.06 | 0.15 ± 0.05 |

Subsamples of the particles were scattered on a slide, flattened by over-laying a piece of glass and images captured with a Zeiss PlanNeoFluar Microscope at 33.5 magnification. The particles analysed were not overlapping and not lying on the edges of the image. Images were analysed using ImageJ software (v. 1.53).

***Kinetic adsorption experiment***

Fig. S4. Changes in phosphorus concentration over time for different solid types with a soid:liquid ratio of 0.2 g : 5mL and 5 mg L^-1^ phosphate solution. PMP = pristine PE, WMP = artificially weathered PE, S1 and S2 are soils, S1+PMP, S1+WMP, S2+PMP and S2+WMP are 50:50 wt % mixtures

***Analytical quality control data***

Table S2. Quality control for chemical analyses of solutions resulting from the different experiments.

| Parameters | Name of experiment | Accuracy (%) | Precision (%) | Detection limit  (mg / L) |
| --- | --- | --- | --- | --- |
| Phosphorus | Kinetic | 98.19 | 0.78 | 0.07 |
| Phosphorus | Adsorption | 98.60 | 2.38 | 0.77 |
| Phosphorus | Desorption | 97.92 | 1.92 | 0.61 |
| Phosphorus | pH | 98.26 | 0.81 | 0.05 |
| Phosphorus | Concentration of background electrolyte | 97.54 | 0.39 | 0.06 |

Detection limits for the analytical instruments used were calculated as the mean plus six times the standard deviation of ten repeated measurements of the blank standard (Walsh, 1997). Accuracy of calibration was determined by analysis of an in-house reference sample (0.5 mg L^-1^ phosphorus solution). Analytical precision was calculated from the coefficient of variation (CV) determined from the duplicate analysis of 10% of the samples that were at least 100 times higher than the detection limit and determining the median of the difference between the duplicate measurements expressed as a percentage of their mean value (Gill and Ramsey, 1997).

Table S3a-d - See Excel files Table S3a.xlsx; Table S3b.xlsx; Table S3c.xlsx, Table S3d.xlsx

Which give Initial adsorption experiment, fixed pH, ionic strength 0.0, 0.01 and 0.1 M NaNO_3_ data and regression fits to linearised isotherms.

***Microplastic ageing results***

Fig S5. Changes in the FTIR spectra of HDPE after exposure to UV radiation with 185 nm wavelength. Exposure duration was four hours per day over a period of 105 days.

***References***

Courchesne F, Turmel, M-C (2008) Extractable Al, Fe, Mn and Si. In: Carter MR and Gregorich EG (Eds) Soil sampling and methods of analysis. Canadian Society of Soil Science. CRC Press, Boca Raton, Florida, USA.

Gill, R. Ramsey, M.H., 1997. What a geochemical analysis means, in: Gill, R. (Ed.), Modern analytical geochemistry: an introduction to quantitative chemical analysis techniques for earth, environment and materials scientists. Longman Geochemistry, UK, pp. 1-11.

Hoogsteen, M.J.J., Lantinga, E.A., Bakker, E.J., Tittonell, P.A., 2018. An evaluation of the loss-on-ignition method for determining the soil organic matter content of calcareous soils. Commu. Soil Sci. Plant Anal. 49, 1541–1552.

Noh, J.S., Schwarz, J.A., 1989. Estimation of the point of zero charge of simple oxides by mass titration. J. Colloid Interface Sci. 130, 157–164.

Rowell, D.L., 1994. Soil Science: Methods and Applications. Prentice Hall, Harlow, UK, pp. 350.

USDA (United States Department of Agriculture), 1951. Soil Survey Manual. Bureau of Plant Industry, Soils and Agricultural Engineering, United States Department of Agriculture, Washington, DC.

Walsh, J.N., 1997. Inductively coupled plasma-atomic emission spectrometry (ICP-AES), in: Gill, R. (Ed.), Modern analytical geochemistry: an introduction to quantitative chemical analysis techniques for earth, environment and materials scientists. Longman Geochemistry, UK, pp. 41-66.
