# Supplementary material for: Polyethylene microplastic can adsorb phosphate but is unlikely to limit its availability in soil
Source: Heliyon. 2023 Dec 17;10(1):e23179. doi: 10.1016/j.heliyon.2023.e23179 (PMC10772576; doi:10.1016/j.heliyon.2023.e23179)
Supplement: Multimedia component 2 [file mmc2.docx]

- Weathered microplastics (MPs) adsorb more phosphate than pristine MP
- Phosphate adsorption to MPs is best described by linear and Freundlich isotherms
- Soils can adsorb more phosphate than MPs
- Phosphate sorption to MP is reversible
- Phosphate sorption by MPs is unlikely to limit P availability or plant growth
